# Acute Cardiac Air Embolism

**DOI:** 10.5811/cpcem.2017.12.36422

**Published:** 2018-01-12

**Authors:** Leslie A. Bilello, Brian Q. Gacioch, James P. Phillips

**Affiliations:** * Harvard Medical School, Beth Israel Deaconess Medical Center, Department of Emergency Medicine, Boston, Massachusetts; † Medical Corps, United States Air Force, Malcolm Grow Emergent Care Center, Joint Base Andrews, Maryland; ‡ George Washington University Hospital, Department of Emergency Medicine, Washington, District of Columbia

## CASE PRESENTATION

**Image 1:**
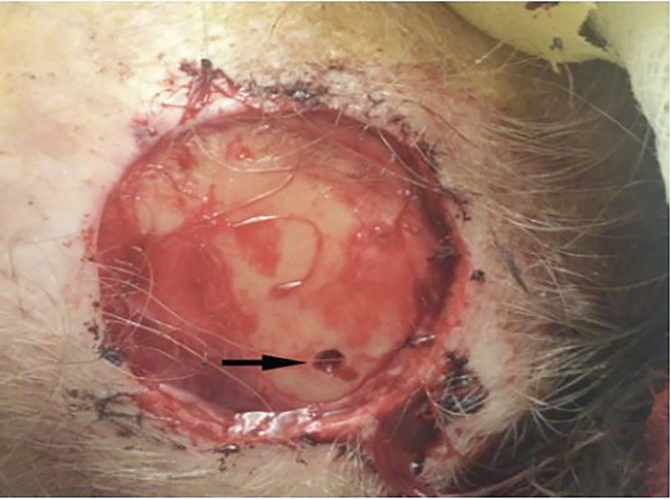
Superficial basal cell carcinoma excision site on the parietal scalp. The procedure also required an area of deep bone curettage (black arrow).

**Image 2:**
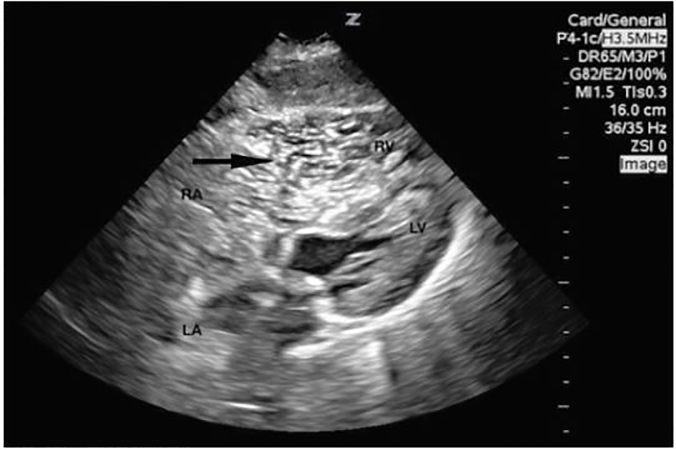
A subcostal cardiac view demonstrated normal left ventricle (LV) contractility, decreased right ventricular (RV) contractility, and RV dilation greater than 1.5 times the LV diameter. Copious hyperechoic mobile bodies were noted within the right atrium (RA) and RV (black arrow). A parasternal short view, not pictured, revealed LV septal in-bowing during systole and diastole. *LA*, left atrium.

An 84-year-old female status post-Mohs micrographic surgery (MMS) presented to the emergency department (ED) for evaluation after a syncopal episode. Surgical excision of a scalp basal cell carcinoma occurred immediately prior to arrival (Image 1). Hemostasis was achieved by both cauterization and direct pressure. Within one minute, patient experienced a 10-second syncopal episode and was hypoxic (64% on room air). The patient arrived via ambulance with blood pressure 108/56 mm Hg and 92% on 15 liters per minute via non-rebreather. Crepitus was appreciated during cardiac auscultation. We performed a focused cardiac ultrasound (Image 2).

## DIAGNOSIS

Point-of-care cardiac ultrasound suggested acute air embolism with right heart strain as the cause of the patient’s syncope. An air embolism is a rare but serious complication of any procedure that may involve venous or arterial vasculature. Air emboli in the setting of MMS has previously been cited in a dermatology case report.^1^ It has also been recorded in head and neck surgery, dental surgery, and is a known complication of seated-position neurosurgical operations.^2^–^5^ Complications include, but are not limited to, coronary or cerebral infarct, complete cardiovascular collapse, and death.^6^

**Image 3:**
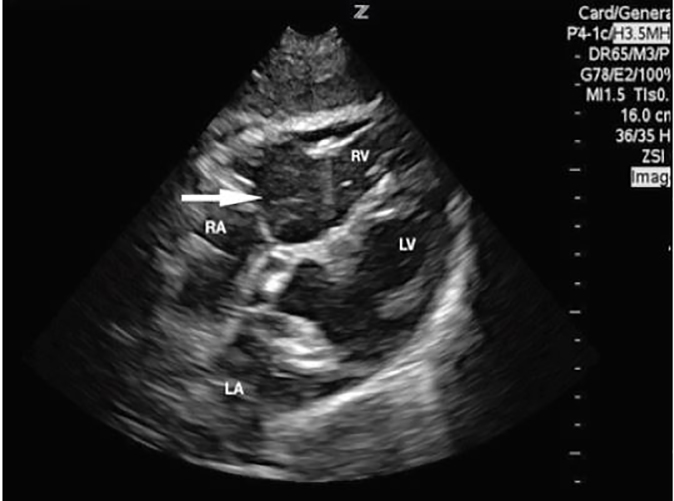
Repeat subcostal view approximately 12 minutes later revealed improved, but not resolved, right ventricle (RV) dilatation, significantly decreased density of air bubbles in the RV (white arrow), trace air bubbles in the left ventricle (LV). At this time, the patient’s vital signs had normalized and her oxygen requirement was significantly decreased. *RA*, right atrium; *LA*, left atrium.

To our knowledge, this is the first case within the ED setting to capture acute air emboli causing hemodynamic compromise on point-of-care ultrasound (POCUS). We placed the patient in reverse Trendelenburg while providing supplemental oxygen which lead to real-time clinical improvement with echocardiographic evidence (Image 3). Computed tomography angiogram of the chest ruled out true pulmonary embolism. This case further demonstrates the value of POCUS as a diagnostic tool in the hemodynamically unstable patient. Although clinically significant air emboli are rare, the need to consider the diagnosis is critical. Recognition should prompt treatment with supine or reverse Trendelenburg positioning while providing supplemental oxygen and consideration of hyperbaric oxygen therapy.^6^

CPC-EM Capsule

What do we already know about this clinical entity?

Acute air emboli may result in coronary or cerebral infarct, cardiovascular collapse and death without appropriate diagnosis and treatment.

What is the major impact of image(s)?

To our knowledge, these are the first ED ultrasound images to capture air emboli causing hemodynamic compromise as well as real-time clinical improvement with echocardiographic evidence.

How might this improve emergency medicine practice?

EM physicians must consider air embolus when approaching a hemodynamically unstable patient, particularly after procedures involving venous or arterial vasculature.
